# Achieving Smart Photochromics Using Water-Processable, High-Contrast, Oxygen-Sensing, and Photoactuating Thiazolothiazole-Embedded Polymer Films

**DOI:** 10.1021/acsaom.4c00014

**Published:** 2024-04-27

**Authors:** Tyler J. Adams, Naz F. Tumpa, Maithili Acharya, Quy H. Nguyen, Nuren Shuchi, Mia Baliukonis, Sarah E. Starnes, Tino Hofmann, Michael G. Walter

**Affiliations:** † Department of Chemistry, University of North Carolina at Charlotte, Charlotte, North Carolina 28223, United States; ‡ Department of Physics and Optical Science, University of North Carolina at Charlotte, Charlotte, North Carolina 28223, United States

**Keywords:** photochromic, photofluorochromic, photoactuator, thiazolothiazole, oxygen sensor, sensing, green chemistry

## Abstract

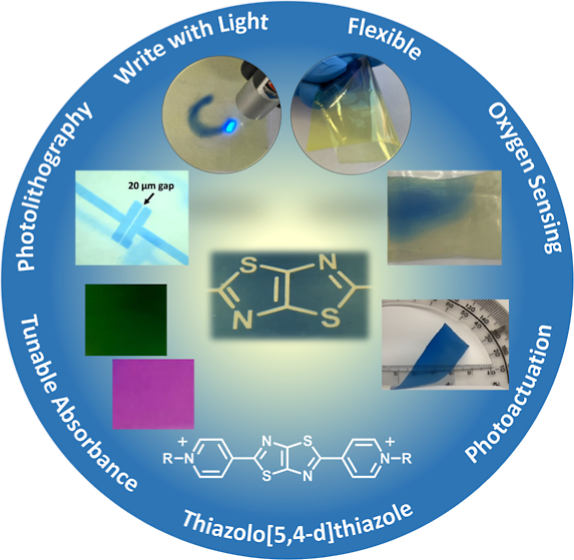

Water-soluble dipyridinium thiazolo[5,4-*d*]thiazole (TTz) compounds are incorporated into inexpensive poly(vinyl alcohol) (PVA)/borax films and exhibit fast (<1 s), high-contrast photochromism, photofluorochromism, and oxygen sensing. Under illumination, the films change from clear/yellow TTz^2+^ to purple TTz^•+^ and then blue TTz^0^. The contrast and speed of the photochromism are dependent on the polymer matrix redox properties and the concentration of TTz^2+^. The photoreduced films exhibit strong, near-infrared light (1000–1500 nm) absorbances in addition to visible color changes. Spectroscopic ellipsometry was used to establish the complex dielectric function for the TTz^2+^ and TTz^0^ states. Incorporating non-photochromic dyes yields yellow-to-green and pink-to-purple photochromism. Additionally, when illuminated, reversible photoactuation occurs, causing mechanical contraction in the TTz-embedded films. The blue film returns to its colorless state via exposure to O_2_, making the films able to sense oxygen and leak direction for smart packaging. These films show potential for use in self-tinting smart windows, eyeglasses, displays, erasable memory devices, fiber optic communication, and oxygen sensing.

## Introduction

Photochromics are versatile material technologies with uses in a wide variety of applications such as self-tinting smart windows, eyeglasses, displays, and glucose sensors.^1−3^ Some materials may also exhibit photofluorochromism, where the fluorescence intensity or wavelength changes with exposure to light,^4,5^ and are useful for displays, erasable memory devices,^6^ or sensors.^7^ Organic materials are especially advantageous for these types of applications because of their high contrast, flexibility, easy processability, and inexpensive starting materials.^8^ A variety of organic materials have been used for photochromic and photofluorochromic devices and films. For instance, organic dyes like Reversacol Berry Red suspended in Paraloid B-72 and polyvinyl butyral polymeric films yield high-contrast photochromism that bleaches with heat.^9^ Photofluorochromism was demonstrated using cyanostilbene derivatives in poly(vinyl alcohol) (PVA) nanowire films.^6^ A variety of processing conditions have been explored such as solvent-free indolinospirooxazine/ethylene-vinyl acetate copolymer or spirooxazine or spiropyran/disentangled ultrahigh-molecular-weight polyethylene in a two-roll mill,^10,11^ and spiropyran-based or spirooxazine-based photochromic compounds dissolved in alcohols slot-die-coated onto poly(ethylene terephthalate) (PET) substrates.^12^

There remain many critical challenges for organic photochromics, including relatively slow switching speeds, limited spectral coverage, poor reversibility, tunability, and integration with existing technologies. Ideally, organic photochromic materials should also be made environmentally friendly using water-based casting for efficient roll-to-roll processing for scale-up purposes. A relatively new family of chromogenic materials that address these criteria are organic materials containing thiazolo[5,4-*d*]thiazole (TTz). Studies of the heterobicyclic TTz moieties have recently gained interest because of their high fluorescence quantum yields and multifunctional chromogenic properties (electrochromism, electrofluorochromism, and photochromism).^8,13,14^ The variety of applications and recent studies is impressive, with TTz-based MOFs for chromogenic applications and sensing,^15,16^ cell membrane voltage-sensitive dyes,^17^ organic photovoltaics,^18^ solvent vapor sensing,^19^ organic field effect transistors,^20−22^ organic light-emitting diodes,^23^ and redox flow batteries.^24^ Recently, we reported a simple, multifunctional electrochromic device using dipyridinium TTz^2+^ derivatives in a PVA/borax hydrogel that exhibited stable/reversible cycling and multiple coloration redox states that were also sensitive to light illumination.^14^

In this work, we expand upon the use of dipyridinium TTz’s and incorporate them into a durable, cross-linked PVA polymer, creating a photochromic, photofluorochromic, photoactuating, and oxygen-sensing PVA/borax polymer film (using 0.4–5.0 wt % TTz). PVA is an inexpensive, commonly used polymer that is a “green polymer” because it can degrade over time and is soluble in water. It can also be blended or copolymerized with other monomers to create copolymers for drug delivery, food packaging, and biomaterials.^25^ We also examine similar TTz/polymer blends using agarose and poly(methyl methacrylate-*co*-methacrylic acid) (PMMA-MAA) films.

## Experimental Section

### Materials and Instrumentation

Dithiooxamide, 4-pyridinecarboxaldehyde, (3-bromopropyl)-trimethylammonium bromide, PVA Mw 11,000–31,000, sodium tetraborate decahydrate (borax), PMMA-MAA Mw 34,000, agarose, methyl *p*-tosylate, hexanes, and dimethylformamide (DMF) were all purchased from Sigma-Aldrich, Ambeed, and Baker Scientific. ^1^H NMR measurements were taken using JEOL 500 MHz NMR and JEOL 300 MHz NMR. Mass spectrometry measurements were obtained with a PerSeptive Biosystems Voyager MALDI-TOF mass spectrometer.

A Varian Cary 50 Bio was used for UV–vis measurements, and a Shimadzu RF-5301PC was used for fluorescence measurements. Near-infrared (NIR) measurements were collected with a Varian Cary 5000.

A Gamry Reference 600 instrument was used for cyclic voltammetry with a saturated calomel electrode reference, a platinum foil counter electrode, and a platinum button working electrode.

The 14 day low-oxygen studies were conducted in an MBraun MB-20G glovebox using an Ocean Optics QE65000 spectrophotometer and an EcoSmart 800 lm multicolor LED light bulb selected to 630 nm red light (Figure S12).

### Ellipsometry Measurements

A commercial spectroscopic ellipsometer (RC2, J.A. Woollam Co., Inc.) equipped with focusing probes was used. The instrument nominally allows data acquisition in a spectral range from 0.5 to 5.9 eV using a broad-band Xe arc lamp. The focusing probes enable the measurement of sample areas with a diameter of approximately 130 μm. To prevent the unintentional photochromic transition of the TTz-embedded polymer sample during the ellipsometric measurements, the instrument is augmented with an edge filter with a cut-on wavelength of 500 nm. The edge filter is attached to the source-side focusing probe, and its optical effects are addressed during the instrument calibration. For the investigation of the optical properties of the TTz^0^ state, a 405 nm laser (13.25 mW mm^–2^) is used for 10 min as an excitation source. A purge chamber is used to maintain a N_2_ atmosphere and avoid oxidation by ambient oxygen. Ψ- and Δ-spectra were obtained in a N_2_ atmosphere in the spectral range from 0.8 to 2.4 eV at three angles of incidence: Φ_a_ = 56, 57, and 58°.

The analysis of the spectroscopic ellipsometry data acquired from the bulk-like TTz-embedded polymer samples was carried out with a commercial software package (WVASE32, J.A. Woollam Co., Inc.). The optical properties of the TTz-embedded polymer for TTz^2+^ and TTz^0^ states are described using a multioscillator model composed of Lorentz oscillators. This approach of using dispersion functions with Lorentz broadening results in a Kramers–Kronig-consistent complex dielectric function and provides sufficient flexibility to accurately describe the experimental data. During the analysis, the oscillator parameters, such as amplitude, resonant energy, and broadening, are varied using a Levenberg–Marquardt algorithm until the best match between the experimental and the model-calculated data is achieved by minimizing a weighted error function χ^2^.^26^

### Synthesis

#### Synthesis of 2,5-Di(pyridin-4-yl)thiazolo[5,4-*d*]thiazole **(Py**_**2**_**TTz)**

Dithiooxamide (1.9916 g, 16.6 mmol) and 4-pyridinecarboxaldehyde (4.4 mL, 46.7 mmol) were refluxed in 60 mL of DMF at 153 °C for 8 h in an aerated environment. The reaction mixture was cooled to room temperature, and the obtained tan precipitate was filtered via vacuum. The solid was then washed with water and dried under vacuum to give a tan solid (3.732 g, 75.9% yield). Molecular characterization data quantitatively matched previously reported values.^13,14,24^^1^H NMR (500 MHz, CDCl_3_), 8.78 (dd, *J* = 1.6, 4.6 Hz, 4H), 7.88 (dd, *J* = 1.6, 4.6 Hz, 4H) ppm. MS [MALDI-TOF]: *m*/*z* calcd for C_14_H_8_N_4_S_2_, 296.376; found, 298.66.

#### Synthesis of *N*,*N*′-Di(trimethylaminopropyl)-2,5-bis(4-pyridinium)thiazolo[5,4-*d*]thiazole **[((NPr)**_**2**_**TTz**^**4+**^**)Br**_**4**_**]**

Py_2_TTz (2.9906 g, 10.1 mmol) was heated with (3-bromopropyl) trimethylammonium bromide (6.5995 g, 25.3 mmol) in 35 mL of DMF under nitrogen at 100 °C for 72 h. The precipitate obtained was vacuum-filtered, rinsed with DMF and acetonitrile, and then dried in the vacuum oven to give a yellow solid (7.2742 g, 87.8% yield). Molecular characterization data quantitatively matched previously reported values.^14,24^^1^H NMR (500 MHz, D_2_O): 2.55 (m, 4H), 3.08 (s, 18H), 3.46 (t, *J* = 8.0 Hz, 4H), 4.67 (t, *J* = 6.5 Hz, 4H), 8.59 (d, *J* = 5.5 Hz, 4H), 8.95 (d, *J* = 5.5 Hz, 4H) ppm.

#### Synthesis of *N*,*N*′-Dimethyl 2,5-bis(4-pyridinium)thiazolo[5,4-*d*]thiazole Ditosylate **[(Me**_**2**_**TTz**^**2+**^**)Tos**_**2**_**]**

Py_2_TTz (0.2891 g, 0.98 mmol) was warmed to 30 °C for 48 h in 10 mL of methyl *p*-tosylate. The precipitate was collected, washed with hexanes, and dried under vacuum to yield 0.5804 g (89% yield) of a brownish yellow solid.^13,14^^1^H NMR (300 MHz, CD_3_CN): 8.74 (d, *J* = 6.87 Hz, 4H), 8.50 (d, *J* = 6.87 Hz, 4H), 7.57 (d, *J* = 7.98 Hz, 4H), 7.12 (d, *J* = 7.98 Hz, 4H), 4.32 (s, 6H), 3.24 (s, 3H) ppm.

### Film Preparation

PVA/borax films were made by dissolving NPrTTz in 4% PVA solution and then adding the appropriate amount of 4% borax solution. Depending on the borax concentration, additional water was added for a thinner consistency for the coating. The solutions were prepared as follows: 5% borax: 60 mL of 4% PVA solution, 10.4 mg of NPrTTz, 3 mL of 4% borax solution, and a 60 μm coater gap; 10% borax: 60 mL of 4% PVA solution, 10.5 mg of NPrTTz, 7 mL of 4% borax solution, 5 mL of DI water, and a 60 μm coater gap; 14% borax: 60 mL of 4% PVA solution, 11.0 mg of NPrTTz, 10 mL of 4% borax solution, 10 mL of DI water, and a 60 μm coater gap. PVA films were made by dissolving 10.0 mg of NPrTTz in 60 mL of 4% PVA solution and coating them with a 60 μm gap. Agarose films were made by dissolving 1.0148 g of agarose and 5.3 mg of NPrTTz in 15 mL of water and then coating (80 μm gap) while warm. The PMMA-MAA film was made by mixing 5.0003 g of PMMA-MAA, 25 mg of Me_2_TTz^2+^ 2Tos^–^, and 10 mL of dichloromethane and coating with a 50 μm coater gap. A LianDu six in. adjustable film coating applicator (Figure S13) was used to coat the films in a doctor-blade-like fashion. The films were coated onto mylar sheets (0.1 mm, 4 mil PET). The film thickness was measured with a digital micrometer, 20–30 μm film thickness.

For differing TTz concentrations, the solutions were prepared as follows: 0.4% NPrTTz: 60 mL of 4% PVA solution, 11.0 mg of NPrTTz, 10 mL of 4% borax solution, 10 mL of DI water, and a 60 μm coater gap; 1.7% NPrTTz: 60 mL of 4% PVA solution, 51.8 mg of NPrTTz, 10 mL of 4% borax solution, 8 mL of DI water, and a 60 μm coater gap; 3.4% NPrTTz: 60 mL of 4% PVA solution, 102.6 mg of NPrTTz, 10 mL of 4% borax solution, 13 mL of DI water, and a 60 μm coater gap; 5% NPrTTz: 60 mL of 4% PVA solution, 150 mg of NPrTTz, 10 mL of 4% borax solution, 13 mL of DI water, and a 60 μm coater gap.

For different colored films, the solutions were prepared as follows. The yellow to green film was 1.7% (w/w) NPrTTz with green food color [yellow 5 (tartrazine 534.3 g/mol) and blue 1 (brilliant blue FCF 792.85 g/mol)] film: 60 mL of 4% PVA solution, 50.7 mg of NPrTTz, 2 drops of green food color, 10 mL of 4% borax solution, 10 mL of DI water, and a 60 μm coater gap. The pink to purple film had 0.8% (w/w) NPrTTz with a 0.2% (w/w) rhodamine B film: 60 mL of 4% PVA solution, 22.6 mg of NPrTTz, 5.70 mg of rhodamine B, 10 mL of 4% borax solution, 10 mL of DI water, and a 60 μm coater gap.

Films were cut to 2 cm × 2 cm squares and taped to the flat plate film sample holder with black electrical tape for UV–vis and fluorescence spectroscopy measurements. The uvBeast V3 flashlight was held 11 cm from the film during photochromic and photofluorochromic testing. At this distance, the flashlight irradiates the film with 0.54 mW cm^–2^ at a 394 nm light (Figure S14).

Photoactuation tests (Figure S8) were performed using 2 cm × 8 cm cut films, with a straight edge and weight holding down 2 cm × 2 cm of the film, allowing the remaining 2 cm × 6 cm to be exposed and free to bend/curl with light irradiation. The uvBeast V3 flashlight was held at a 45° angle and 11 cm from the film for photoactuation illumination. The photoactuation film in Figure 6e was overall 4 cm × 6 cm with 1 cm × 4 cm fingers cut.

**Figure 1:**
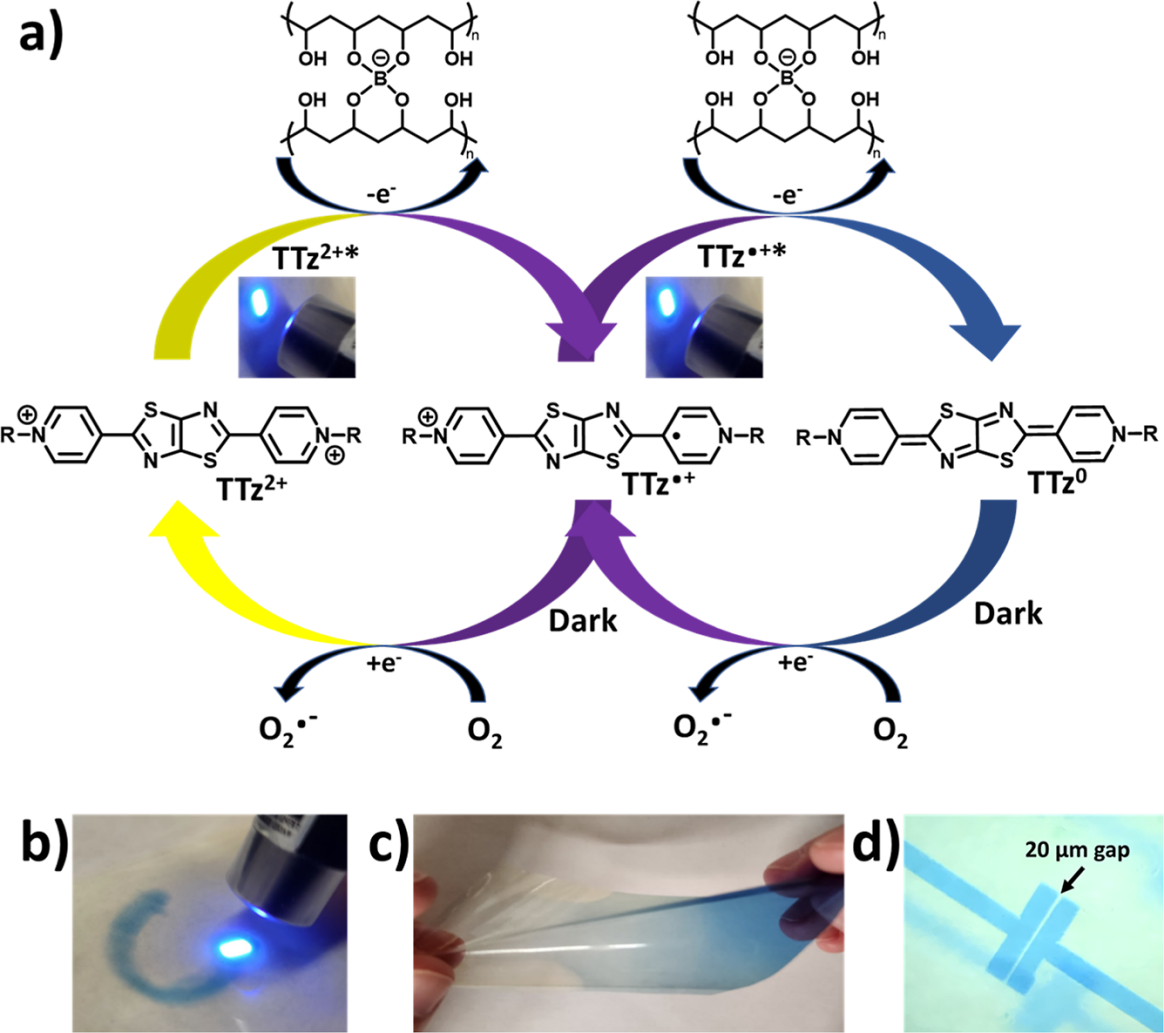
(a) TTz reductions via photoinduced electron transfer and interaction with the cross-linked PVA/borax polymer matrix and reversal when exposed to oxygen; (b) photochromic writing with a 405 nm laser pointer (1–5 mW); (c) free-standing, photochromic, flexible film (∼25 μm thickness) exhibiting illumination-dependent color contrast; and (d) film photolithography using a 20 μm gap mask and a 0.54 mW cm^–2^, 394 nm LED.

**Figure 2:**
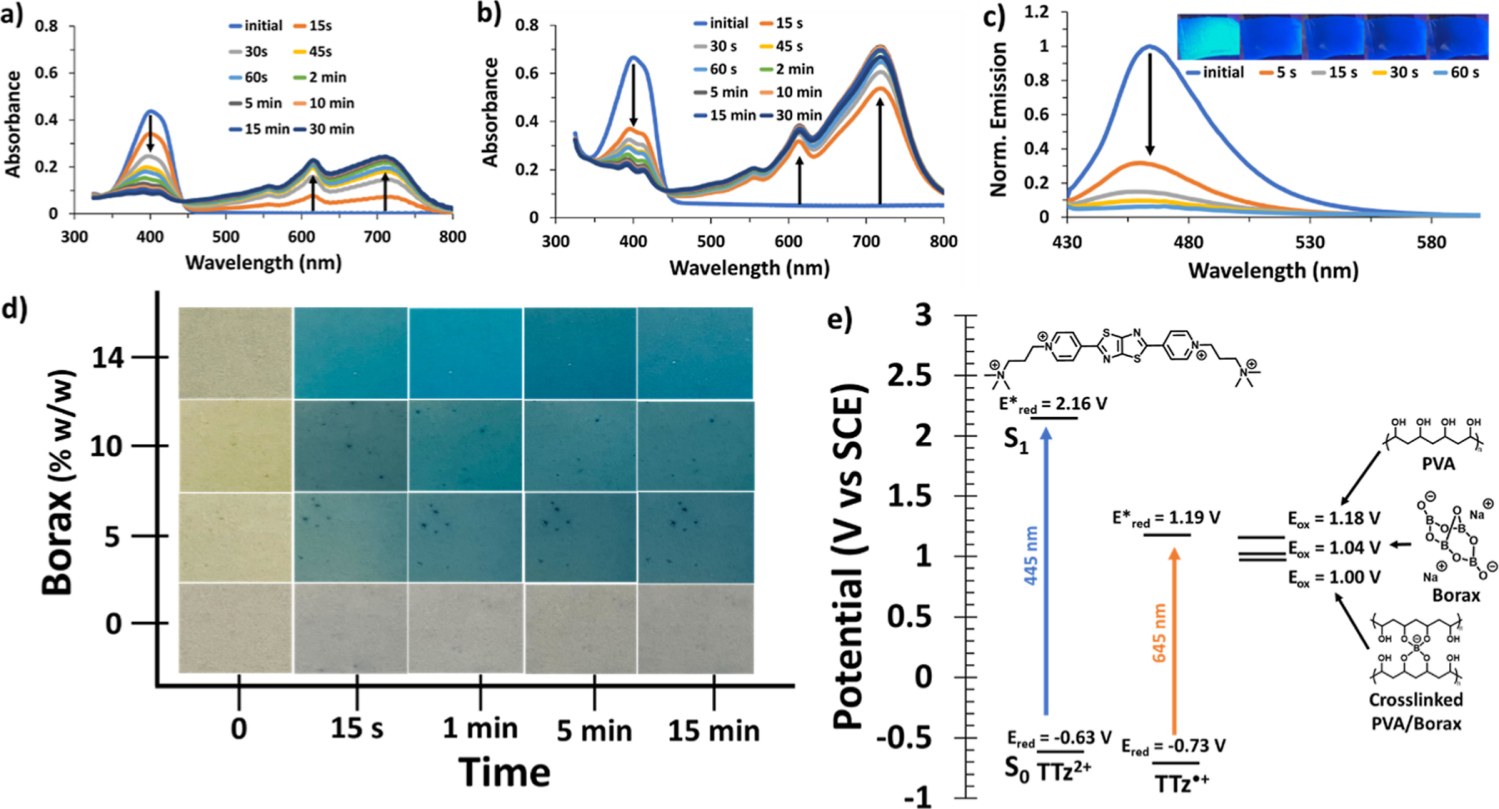
Photochromism of 0.4% (wt %) TTz PVA/borax films with 394 nm irradiation and varying borax cross-linker concentrations: (a) 0% borax; (b) 14% borax; (c) photofluorochromism of 14% borax PVA/borax film (420 nm excitation), with the inset visual representation; (d) visual representation of photochromism; and (e) redox potential diagram of TTz, PVA, borax, PVA/borax mixture, and gelled cross-linked PVA/borax.

**Figure 3:**
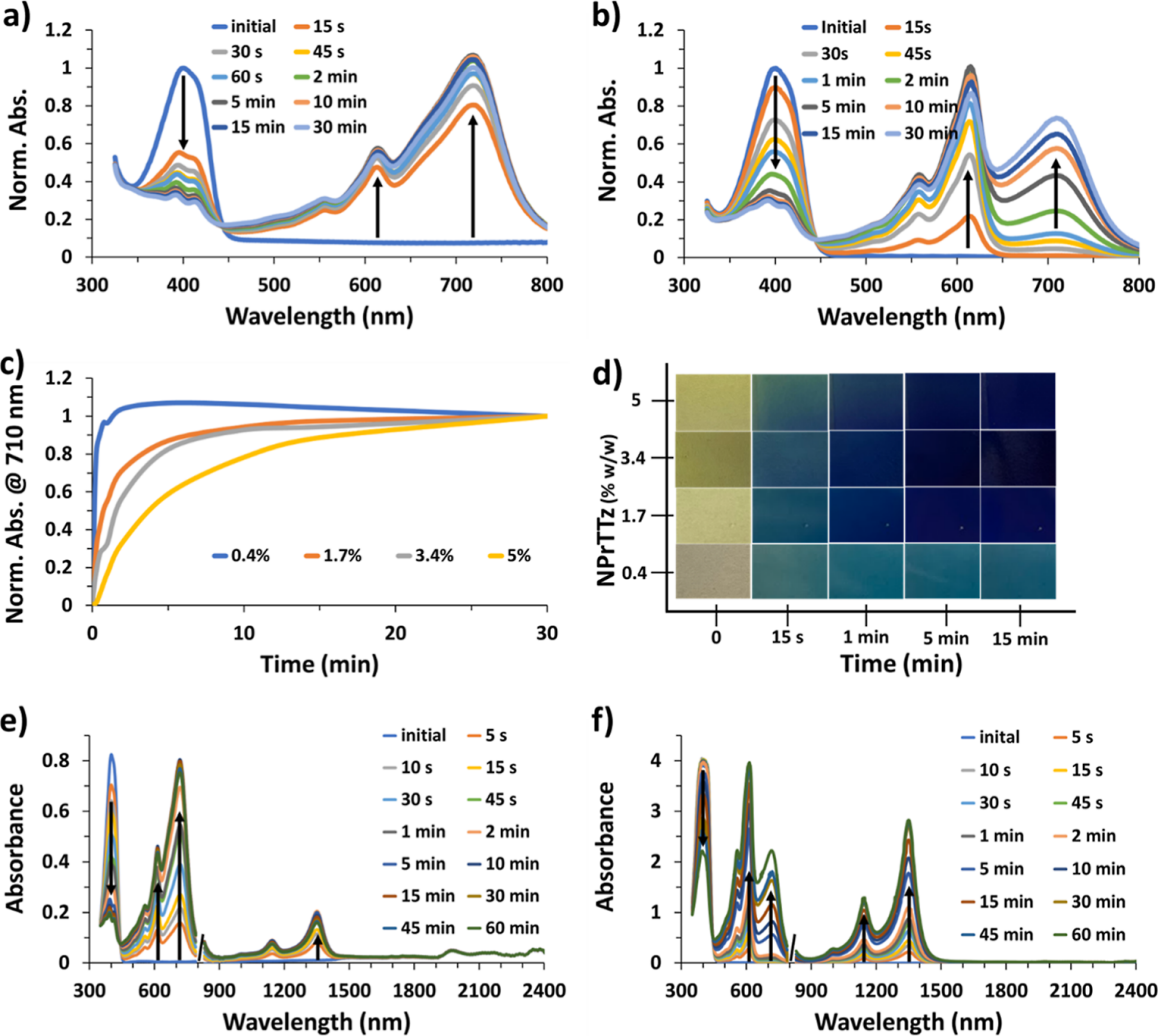
Photochromism of PVA/borax films (14 wt % borax) with different TTz concentrations: (a) 0.4%, (b) 5%, (c) change in 710 nm absorbance over photochromism time, (d) visual representation of photochromism, (e) visible/NIR absorbance of the 0.4% TTz film, and (f) visible/NIR absorbance of the 5% TTz film (wt %).

**Figure 4:**
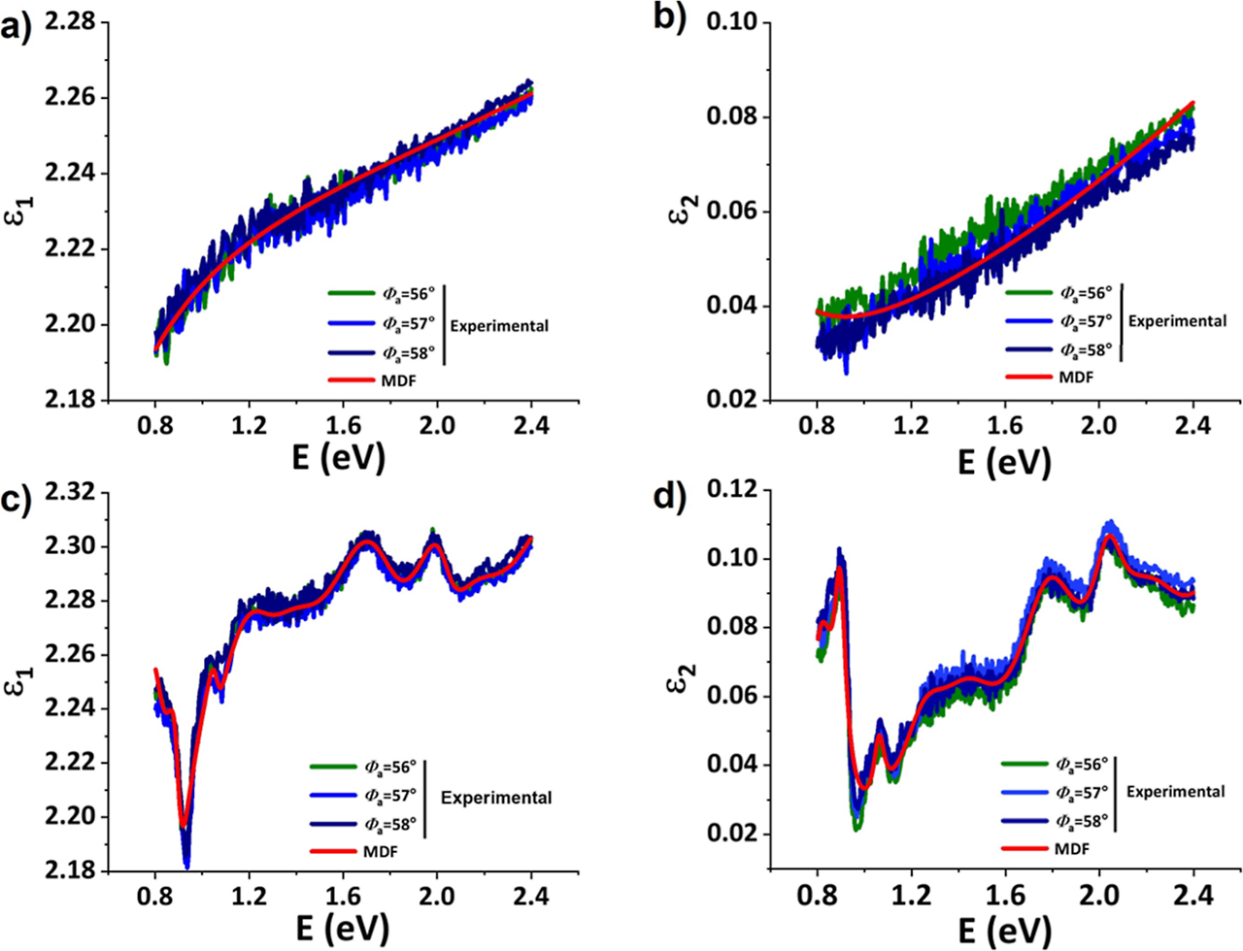
Comparison of the point-by-point inverted complex dielectric function obtained from ellipsometric measurements at three different angles of incidence: Φ_a_ = 56, 57, and 58° with a Lorentz-oscillator-based MDF for a 3.4% TTz-embedded polymer sample. The real ε_1_(*E*) and imaginary ε_2_(*E*) parts of the complex dielectric function of the TTz^2+^ state are shown in parts (a,b), respectively. The corresponding data for the TTz^0^ state are shown in (c,d), respectively [TTz^0^ was generated by illumination with a 405 nm laser (13.25 mW mm^–2^) for 10 min].

**Figure 5:**
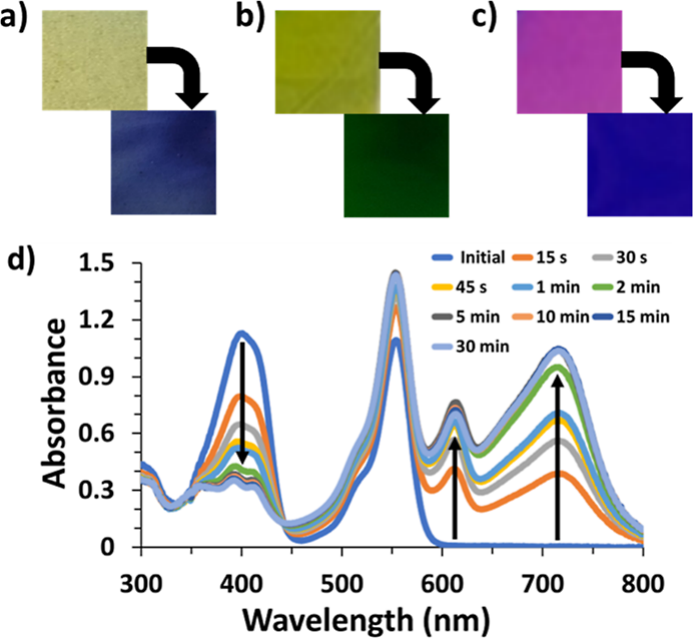
Photochromism of the (a) TTz (1.7%) PVA/borax film, (b) green food color (tartrazine and brilliant blue FCF) and TTz (1.7%) PVA/borax film, (c) rhodamine B (0.2%) and TTz (0.8%) PVA/borax film, and (d) absorbance spectra of the rhodamine B (0.2%) and TTz (0.8%) PVA/borax film.

**Figure 6:**
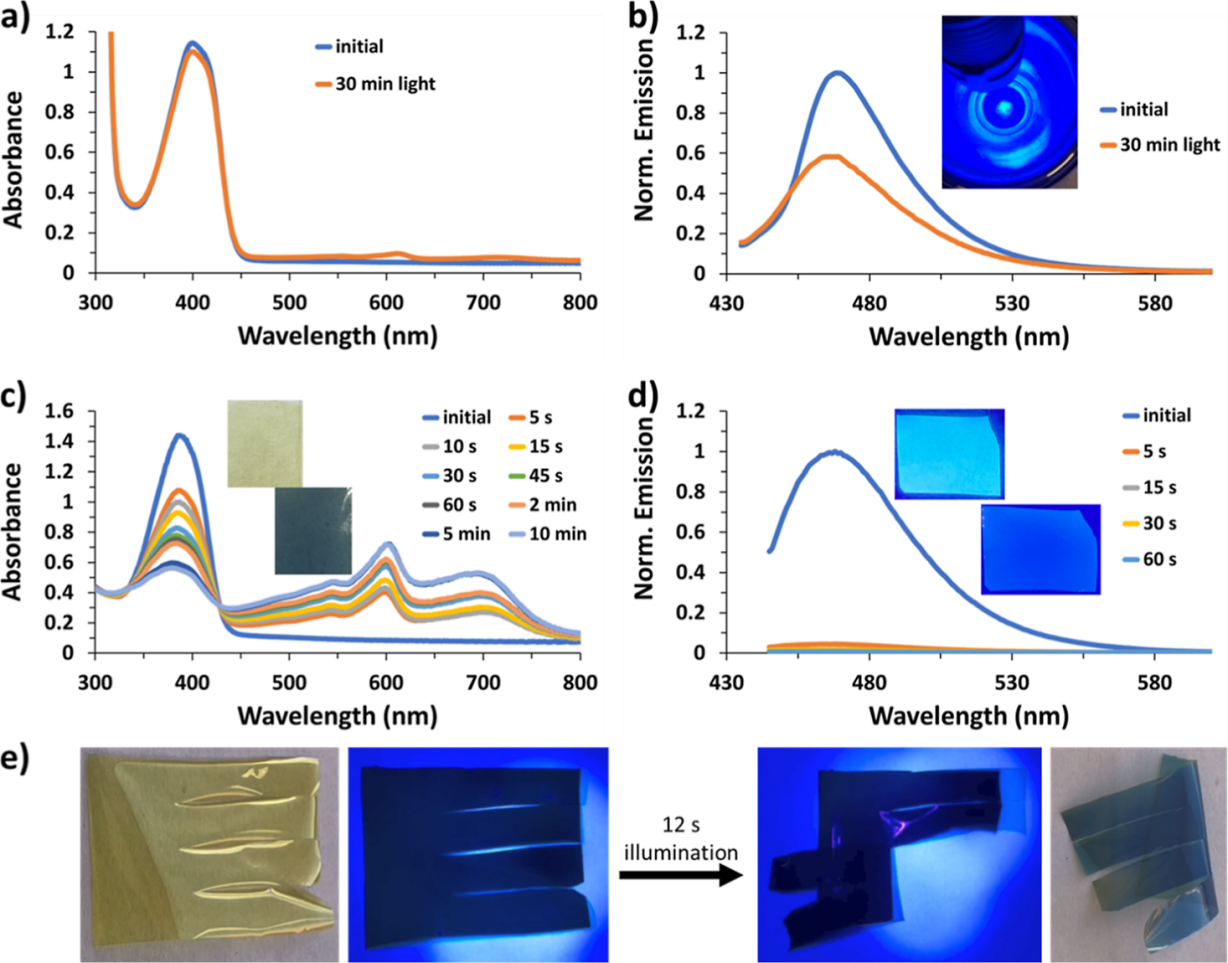
(a) Absorbance and (b) emission of the 0.4% TTz 14% borax film before and after 30 min of illumination while immersed in liquid nitrogen (inset: an image of film fluorescence submerged in liquid N_2_), (c) photochromism (inset: images of the agarose film before and after 1 min illumination), (d) photofluorochromism of the 0.5% TTz agarose film (inset: images of the agarose film before and after 1 min illumination), and (e) photoactuation of free-standing TTz (5%)/PVA before, during, and after light (394 nm) irradiation (film cut into strips).

**Figure 7:**
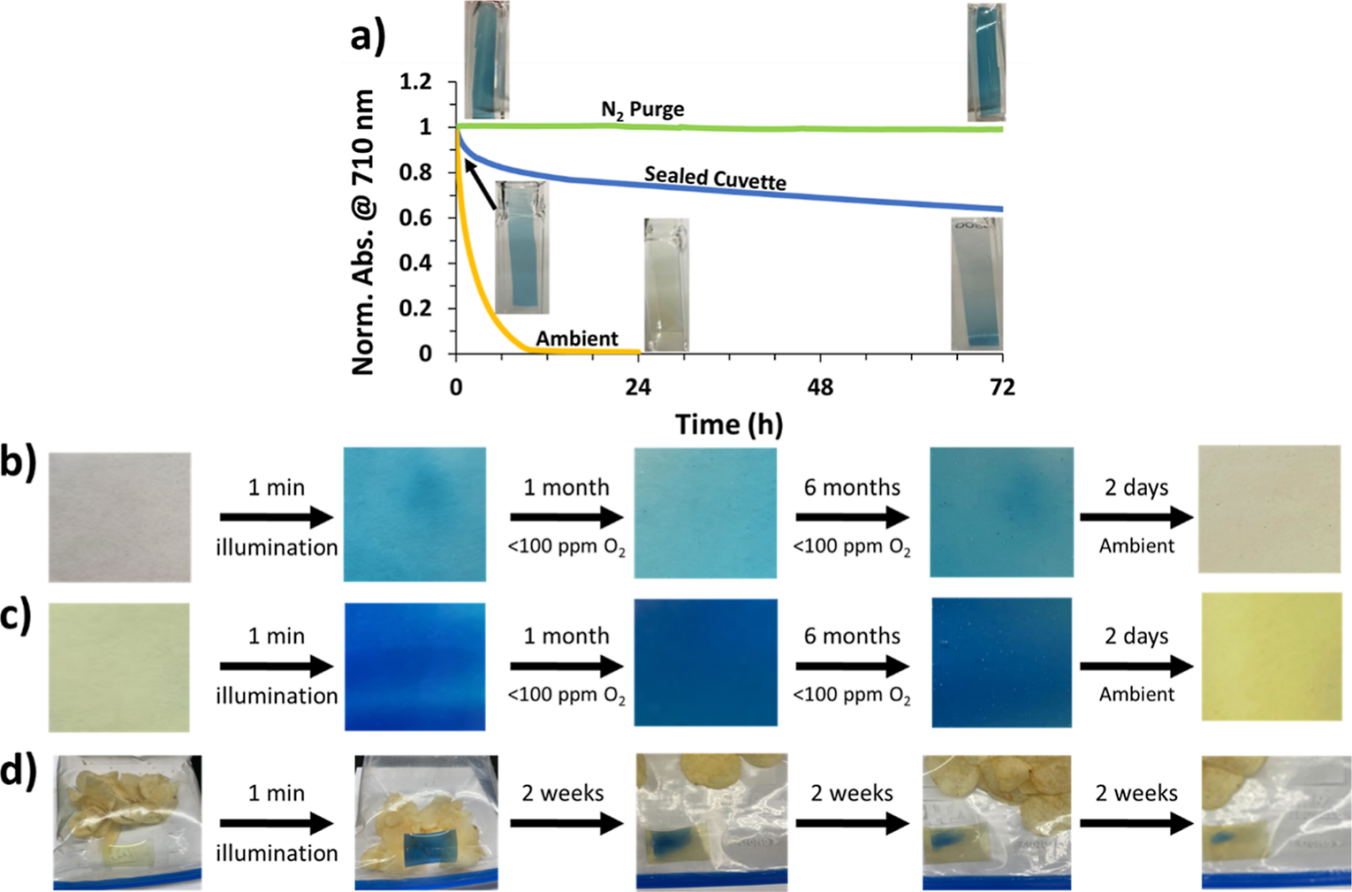
(a) Change in the 710 nm absorbance over time at low ppm of O_2_ levels and ambient conditions showing oxygen sensitivity, with corresponding images; (b) photoactivated 0.4% TTz film in a glovebox atmosphere; (c) photoactivated 5% TTz film in a glovebox atmosphere; and (d) oxygen-sensing TTz film in a nitrogen-flushed zipper bag.

The pictures of photochromism (Figures 2d, 3d, 5a–c, 6c, and 7b,c) were obtained using white paper as a background to show the color change contrast and transparency of the films. The TTz-embedded films were cut to approximately 5 cm × 5 cm for visual representation. Pictures of photofluorochromism were obtained in a similar manner, with a black background and approximately 3 cm × 3 cm cut film, as shown in Figures 2c and 6d.

## Results and Discussion

The TTz/PVA films address many of the challenges faced with organic photochromics. Most notably, they show reversible, fast, and high-contrast photochromic/photofluorochromic shifts utilizing low-cost materials and minimal processing steps. In addition, the inexpensive films provide a broad spectral color change with absorbance changes extending into the NIR. The absorbance of NIR light is advantageous for applications like photonics and telecommunications (fiber optics utilize the 1310 and 1550 nm light),^27^ as well as organic photovoltaics and semitransparent, high-efficiency solar windows.^28^ Materials that absorb NIR light can also be used as window glazing that rivals current low-e coatings for more energy-efficient buildings by reducing solar heat gain.^29^ Using molecular systems that absorb NIR light is also useful for photothermal conversion and photothermal therapy.^30^ Lastly, the new TTz/PVA photochromic polymeric materials presented in this report show unique oxygen sensing capabilities. Color-changing oxygen-sensing materials have a wide variety of uses for smart packaging,^31,32^ medical bandages,^33^ and wearable devices for confined spaces.^34^ Food is commonly packaged under nitrogen or carbon dioxide to reduce oxygen content to 0.5–2%, which decreases spoilage.^32^ Oxygen-induced spoilage occurs from aerobic microorganism growth, oxidation of oils or lipids, or enzymatic reactions that cause fruit/vegetable browning.^31,32^ Packaging under inert atmospheres is also important for electronics, medical equipment, and pharmaceuticals to prohibit oxidation.

The TTz/PVA materials developed and presented here show photochromic on/off switching that is driven forward by the photo-oxidation of crossed-linked PVA by the TTz^2+^ excited state, allowing for nearly instantaneous color changes (depending upon the excitation intensity). The reverse color change (to yellow/colorless) is driven by the reaction of the reduced TTz dyes with molecular oxygen. Therefore, the speed of both forward and reverse coloration (and fluorescence) can be finely tuned, independent of one another, using the concentration and ratios of TTz and PVA/borax and controlling the diffusion of O_2_ through the polymer film. Figure 1a illustrates the reversible photochromism of the TTz-embedded PVA/borax polymer materials. When the colorless TTz^2+^ is excited by light, photo-oxidation of the cross-linked PVA/borax occurs, and the reduced TTz^•+^ forms. Subsequent reduction of TTz can occur via further photo-oxidation of PVA/borax to form the neutral TTz^0^ (Figure 1a). TTz^0^ can be oxidized back to TTz^2+^ upon exposure to oxygen. Therefore, the flexible photochromic film is activated by light, changing from colorless/yellow film to blue, and by using a laser, a photomask, or a stencil, a design can be made onto the film with high contrast (Figure 1b–d and Videos S1 and S2).

### Borax Cross-Linking Dependence

The addition of a cross-linking additive (borax) into a PVA/TTz solution thickens the polymer solution, forming a hydrogel, and results in better adhesion when coating onto a substrate. After blade coating onto a PET substrate and drying the solution, the resulting TTz film can be peeled off (Figure S13). The initial yellow film containing TTz^2+^ absorbs at 400 nm; however, when illuminated, the TTz reduces to TTz^•+^ absorbing at 610 nm and TTz^0^ absorbing at 710 nm.^13^ The rate of photochromism changes dramatically when comparing the TTz/PVA-only film to a film with 14% (wt %) borax (Figure 2a,b). The primary absorbance at 710 nm increases rapidly with 14% borax, whereas the absorbance of the PVA-only film showed a lower reduced TTz^0^ concentration. Additional TTz/PVA films were tested to confirm that the formation of the fully reduced TTz^0^ was borax concentration-dependent. With increasing borax concentration, the intensity of the 710 nm absorbance increases rapidly (<5 s) and the rate of color change also increases, indicating that the presence of a borax cross-linker enhances the speed of the embedded TTz dye reduction (Figures 2a,b,d and S1). Cyclic voltammetry of the PVA and borax indicates that the irreversible oxidation of the polymer becomes easier once cross-linked with borate (Figure S2). The onset potential for PVA alone is 1.18 V vs SCE but decreases to 1.04 V vs SCE when borax is introduced and 1.00 V vs SCE upon cross-linking. This increasing ease of oxidation is shown and compared to the excited-state redox potentials of TTz in Figure 2e. The lower oxidation potential makes the photoinduced electron transfer more favorable. Although the TTz^2+^ state is highly fluorescent, TTz^•+^ and TTz^0^ are non-emissive, which results in photofluorochromism. The photofluorochromism occurs quickly, starting with <5 s of light exposure (Figure 2c). After 1 min of illumination, the fluorescence drops to 88, 89, 90, and 94% for the 0, 5, 10, and 14% borax concentrations, respectively (Figure S3).

### Effects of the TTz Concentration

In the PVA/borax films with 14% borax, various TTz concentrations were tested. The 0.4% TTz film showed immediate 2e^–^ reduction to the TTz^0^ state, whereas the higher TTz concentration films show slower/stepwise reductions (Figures 3a–d and S4), resulting in the presence of a mix of both TTz^•+^ and TTz^0^ states. The 3.4 and 5% TTz films show that the TTz^2+^ (400 nm absorbance) reduction to TTz^•+^ (610 nm absorbance) occurs prior to the reduction to the TTz^0^ state (710 nm). The rate of TTz reduction is shown in Figure 3c, which compares the onset speed of the 710 nm absorbance (TTz^0^). The 0.4% TTz film reduces rapidly to primarily the TTz^0^ state, whereas 5% TTz shows the presence of both TTz states even after 30 min of light exposure. As expected, the higher concentrations yield much darker films when reduced (Figure 3d).

The colorless/yellow TTz^2+^ state shows only the absorbance at 400 nm, with no additional absorbances from 500 to 2500 nm. When TTz^•+^ is formed via illumination, in addition to the 610 nm absorbance, strong absorbances in the NIR at 1150 and 1350 nm are observed. When analyzing the 0.4% TTz film (Figure 3e), both the first (610 nm) and second (710 nm) reductions occur quickly, and the NIR absorbance (1150 and 1350 nm) increases steadily. However, after the 1350 nm peak maximizes at 2 min of illumination, the absorbance steadily decreases upon further illumination. In addition, the TTz^0^ 710 nm peak continued to increase until 10 min of light exposure. With the higher concentration of TTz embedded in the film (5%) (Figure 3f), the 1350 nm peak increased at a similar rate to the 610 nm TTz^+^ absorbance before the 710 nm absorbance appeared. With continued illumination, all absorbances increased, indicating that the NIR absorbances at 1150 and 1350 nm are associated with the electronic transitions of the radical cation (TTz^•+^). The observed NIR absorbances overlap with the 1310 nm light used for fiber optic communications,^27^ and 1000–1350 nm is biologically relevant for photothermal therapy.^30^

### Complex Dielectric Function

The TTz-embedded polymers reported here show great potential to be integrated in optically tunable devices including tinted lenses, smart windows,^1^ optically rewritable data storage,^6^ optical switching, actuators,^35^ tunable filters,^36^ sensors,^7^ and holographic gratings^36^ due to their high-contrast, fast, and reversible photochromic/photofluorochromic shifts. Accurate knowledge of the complex dielectric function is essential for the design and fabrication of TTz-based tunable optical devices. We recently reported on the complex dielectric function of a solution-processable, non-photochromic TTz derivative.^37^

The complex dielectric function of the bulk-like TTz-embedded polymer was extracted using a numerical wavelength-by-wavelength inversion of the experimental Ψ(*E*) and Δ(*E*) data. For a bulk sample with no surface layers, this approach dispenses with the need for a dielectric function model; however, Kramers–Kronig consistency is not ensured.^38^ Therefore, the complex dielectric function obtained using numerical inversion is compared with a model dielectric function (MDF) composed of eight Lorentz-broadened oscillators obtained through best-fit analysis of the experimental data, as shown in Figure 4.

Figure 4 shows a comparison between the real ε_1_ and imaginary ε_2_ parts of the point-by-point inverted complex dielectric function for the TTz^2+^ and TTz^0^ states for three different angles of incidence: Φ_a_ = 56, 57, and 58° with a best-fit MDF. A very good agreement can be observed between the point-by-point inverted complex dielectric function and the MDF best-fit for the TTz^2+^ and TTz^0^ state data. The dispersion seen in ε_2_ of the TTz^2+^ state (Figure 4b) is indicative of absorption in the long- and short-wavelength regions outside the measured spectral range. The photochromic TTz-embedded polymer in its TTz^0^ state shows strong absorption bands at approximately 0.8, 1, 1.8, and 2.1 eV (illustrated in Figure 4d). Additionally, two broad absorptions are observed within the range of 1.2 and 1.8 eV. The ability to manipulate the photochemical and optical properties of these TTz-embedded polymers through optical stimulation facilitates their integration in externally driven optically tunable smart optical and electronic devices. With accurate knowledge of complex dielectric functions, the design of TTz-based, novel optically tunable devices is now feasible.

### Dual Chromatic TTz/Polymer Blends

Non-photochromic dyes can be incorporated into the films to tune the absorbance characteristics of the film before and after illumination. As shown in Figure 5a–c, the yellow to blue film can be changed to yellow to green with the inclusion of green food dye (tartrazine and brilliant blue FCF), and the addition of rhodamine B affords a pink to purple color change. Figure S5 shows continued 400 nm absorbance during the photochromic absorbance change of TTz, contributing to the green color. Similarly, Figure 5d shows the photochromic absorbance spectra of the rhodamine B/TTz film, which initially has 400 and 550 nm absorbances for TTz^2+^ and rhodamine B, respectively. As TTz^2+^ transitions to TTz^0^, the 400 nm absorbance decreases as the 610 and 710 nm absorbances increase, causing the purple color. The intensity of the 550 nm peak increases only slightly because of the TTz^•+^ absorbance overlapping with the rhodamine B absorbance. In these cases, the green food dye and rhodamine B are not photochromic; they only add an absorbance peak to change the visual color of both the non-illuminated and illuminated portions of the films.

To provide further evidence of the proposed photoinduced electron transfer mechanism resulting in the color change of the TTz embedded films, samples were submerged in liquid nitrogen and illuminated for 30 min. Initially, the films showed the bright fluorescence of TTz^2+^ with no evidence of photochromism. Eventually, the photo-oxidation is observed to proceed very slowly. The absorbance and fluorescence spectra in Figure 6a,b indicate that 30 min of illumination is required to achieve similar color changes that occur after 5 s at room temperature. The photoinduced electron transfer is slowed considerably, as would be expected from a reduced rate of electron transfer from cross-linked PVA to the photoexcited TTz^2+^ under low-temperature conditions. Interestingly, this trend was also observable when a TTz/PVA film was dried under vacuum, resulting in a slightly slower TTz reduction (Figure S6). This suggests the possibility of humidity sensing capabilities using the dried hydrogel/TTz film with photochemically driven color changes that are sensitive to the hydration of the film.

In addition to PVA/borax, films were made using agarose and PMMA-MAA. Agarose, like PVA/borax, is a hydrogel and contains alcohol groups that can be oxidized by photoactivated TTz. Figure 6c,d shows how the agarose film is photochromic, showing the TTz^•+^ state at 600 nm and the TTz^0^ state at 710 nm. Although the absorbance intensities are not as high as those observed in PVA/borax films, the photofluorochromism was nearly instantaneous, and the fluorescence turned off by 95% in <5 s of illumination. Interestingly, a PMMA-MAA/TTz film cast from dichloromethane and shown in Figure S7 does not exhibit photochromism or photofluorochromism. The absorbance spectra show no indication of TTz^•+^ or TTz^0^ at the 610 or 710 nm regions, respectively. Instead, TTz degrades with prolonged illumination, which is indicated by the loss of 372 nm absorbance and fluorescence emission intensity. Since the PMMA-MAA polymer backbone does not include oxidizable functional groups (alcohols/cross-linked PVA/borax), photochromism resulting from the PVA/borax photo-oxidation cannot be observed. These observations again support the proposed mechanism of photoinduced electron transfer in the PVA/borax cross-linked polymer/TTz^2+^ photochromic films.

Interestingly, when the PVA/borax or agarose TTz films are exposed to light, they show photoactuation and bend while changing from colorless/yellow TTz^2+^ to blue TTz^0^. As the films are illuminated, they mechanically contract toward the light, regardless of how they were originally coated (Figure 6e and Video S3). Films with increased TTz content bend faster and more drastically and are more sensitive to light, which is graphically demonstrated in Figure S8. As expected, the photoactuation is thickness-dependent, where thinner films (approximately 20–30 μm) bend faster than thicker films (53 μm). The photoactuation is reversible, and the film flattens when light is taken away. Films without TTz do not contract, and bending does not occur. In addition, testing PVA/borax films without TTz indicates that the ∼2 °C heat increase resulting from the blue LED light source does not contribute to the film contraction/bending. Photoactuating organic films have been previously reported utilizing spiropyran nanocomposite films.^39^ Many researchers have used bilayer systems to create bimorph soft actuators that are highly controlled and light-driven for bioinspired soft robotics, artificial muscles, or photoswitches, which can be compact, easily portable, and miniaturized.^35^ The movements can consist of bending, twisting, oscillating, stretching, or expanding.^35^

### Oxygen Sensing

The PVA/borax films return to the colorless/yellow TTz^2+^ state from the blue TTz^0^ via interaction with oxygen, giving the films the ability to sense oxygen and give a visual indication of the oxygen concentration. Figure 7a shows the initial high absorbance of 710 nm light after illumination and demonstrates how the film’s absorbance changes depending on its environment. When in an open container, the film returns to its colorless/yellow state within 12 h, while if the film is put in a low (sub 100 ppm) oxygen-sealed cuvette, it takes over 72 h to return to the colorless/yellow state. This indicates the slow leakage of oxygen, even in the tightly sealed cuvette. If the cuvette is continuously purged with nitrogen gas to ensure minimal oxygen, the film stays permanently blue. Pictures of the film in the cuvette (Figure 7a) indicate that the film nearest to the cap was colorless, while the bottom was still blue, demonstrating how the TTz film also indicates the direction of oxygen leakage. Materials that can not only sense the presence of oxygen but also indicate the direction or location of a leak are advantageous to eliminate leaks or failure points. To verify the sensitivity, a similar experiment was conducted, monitoring the absorbance change from TTz^0^ to TTz^2+^ while in a ∼100 ppm of O_2_ nitrogen atmosphere (Figure S9). The absorbance did not decrease (indicating the presence of TTz^2+^); instead, the overall absorbance at 630 nm slightly increased over the 14 days. Although the measurement was shielded from light, small amounts of ambient light may have further reduced the TTz film. To visually monitor longer-term color change, two films (0.4% TTz and 5% TTz films) were kept in a low-oxygen glovebox atmosphere for 7 months and showed very little visible color change after being photoactivated (Figure 7b,c). After 7 months, the films were taken out of the glovebox and exposed to ambient O_2_, where they quickly returned to yellow within 2 days. This indicates successful oxygen sensing after 7 months of activated, blue color in low-oxygen environments, which is ideal for food or other products packaged for a 6 month shelf life. The color change is reversible but loses contrast with repeated usage, as shown in Figure S10 as well as Videos S1 and S2.

In the food packaging industry, a vast number of products are sealed under nitrogen. To mimic this, a film was placed in a nitrogen-flushed, zipper-closed food storage bag to show long-term oxygen leakage (Figures 7d and S11). Not only did it indicate the presence of oxygen after 2 weeks, but it also showed what direction the leak was coming from, in this case, the corner of the bag near the zipper. These results show that the TTz films are sensitive to oxygen exposure and yield clear, high-contrast visual indications that can be used for smart packaging and other oxygen-susceptible applications.

## Conclusions

Water-soluble dipyridinium thiazolothiazole compounds incorporated into inexpensive PVA/borax films exhibit fast (<1 s), high-contrast photochromism, photofluorochromism (up to 94%), and oxygen sensing. When exposed to light, the films change color from colorless/yellow TTz^2+^ to purple TTz^•+^ and then blue TTz^0^. The contrast and speed of the photochromism are dependent upon the polymer matrix, how easily it can be oxidized, and the concentration of photoactive TTz. In addition to visible light absorbance, the films also absorb strongly in the NIR at 1150 and 1350 nm. The complex dielectric function of the TTz-embedded polymer was measured with ellipsometry, which indicates strong changes between the TTz^2+^ and TTz^0^ states and suggests the possibility of developing optically tunable devices using these dynamic materials. The addition of non-photochromic dyes can yield additional film color changes, including yellow to green and pink to purple. When the films are illuminated, reversible photoactuation occurs, causing the films to mechanically contract. The blue film returns to its colorless/yellow state via oxidation of TTz^0^ when exposed to O_2_, transforming the films into light-activated oxygen sensors that can also sense leak direction. These films show potential for use in self-tinting smart windows, eyeglasses, displays, erasable memory devices, fiber optic communication, smart packaging, and oxygen sensing.

## Supporting Information Available

Additional experimental UV/vis, fluorescence, and photoactuation figures (PDF)
Small-film photochromism with a stencil (MOV)
Large-film photochromism with a reverse stencil (MOV)
Photoactuation (MOV)

## Supplementary Material

ot4c00014_si_001.pdf

ot4c00014_si_002.mov

ot4c00014_si_003.mov

ot4c00014_si_004.mov
