# Supplementary material for: Achieving Smart Photochromics Using Water-Processable, High-Contrast, Oxygen-Sensing, and Photoactuating Thiazolothiazole-Embedded Polymer Films
Source: ACS Appl Opt Mater. 2024 Apr 27;2(5):704–13. doi: 10.1021/acsaom.4c00014 (PMC11129348; doi:10.1021/acsaom.4c00014)
Supplement: Supplementary file 1 — ot4c00014_si_001.pdf [file ot4c00014_si_001.pdf]

Supporting Information

Achieving Smart Photochromics using Water-Processable, High Contrast, Oxygen Sensing, and Photoactuating Thiazolothiazole-Embedded Polymer Films

*Tyler J. Adams,[1] Naz F. Tumpa,[1] Maithili Acharya,[1] Quy H. Nguyen,[1] Nuren Shuchi,[2] Mia Baliukonis,[1] Sarah E. Starnes,[1] Tino Hofmann,[2] Michael G. Walter[1]**

[1]Department of Chemistry, University of North Carolina at Charlotte, Charlotte, North Carolina 28223, United States

[2]Department of Physics and Optical Science, University of North Carolina at Charlotte, Charlotte, North Carolina 28223, United States

*email: Michael.Walter@charlotte.edu

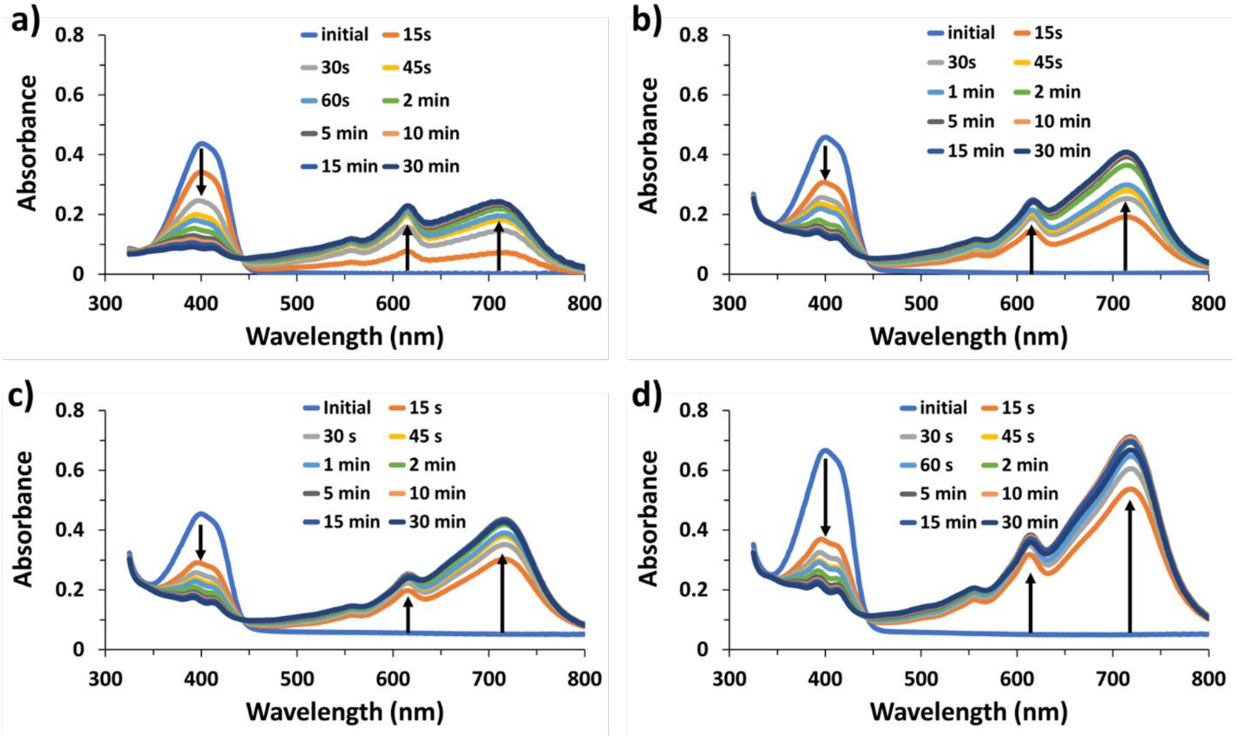

Figure S1: Photochromism of 0.4% (wt. %) TTz PVA/Borax films with different borax concentrations, a) 0% borax, b) 5% borax, c) 10% borax, d) 14% borax.

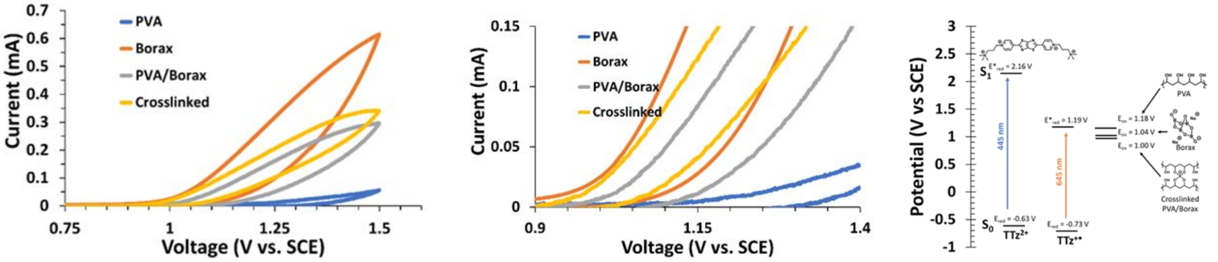

Figure S2: Cyclic voltammetry and corresponding energy level diagram of PVA, borax, PVA/borax mixture, and gelled crosslinked PVA/borax.

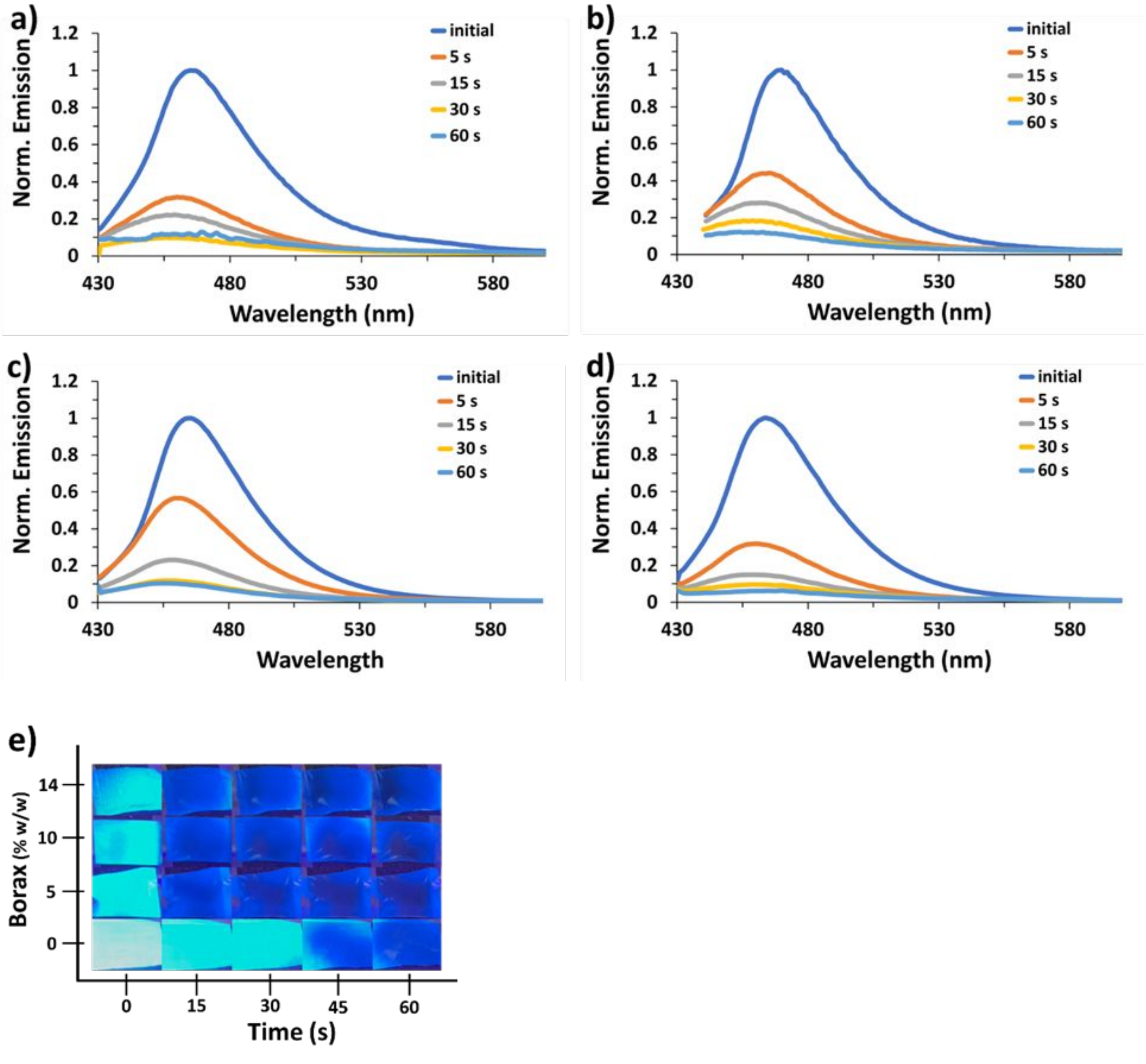

Figure S3: Photofluorochromism of PVA/Borax films with different borax concentrations (excitation/emission), a) 0% (wt. %) borax (418 nm/465 nm), b) 5% borax (425 nm/470 nm), c)

10% borax (415 nm/465 nm), d) 14% borax (420 nm/464 nm), e) visual representation of photofluorochromism.

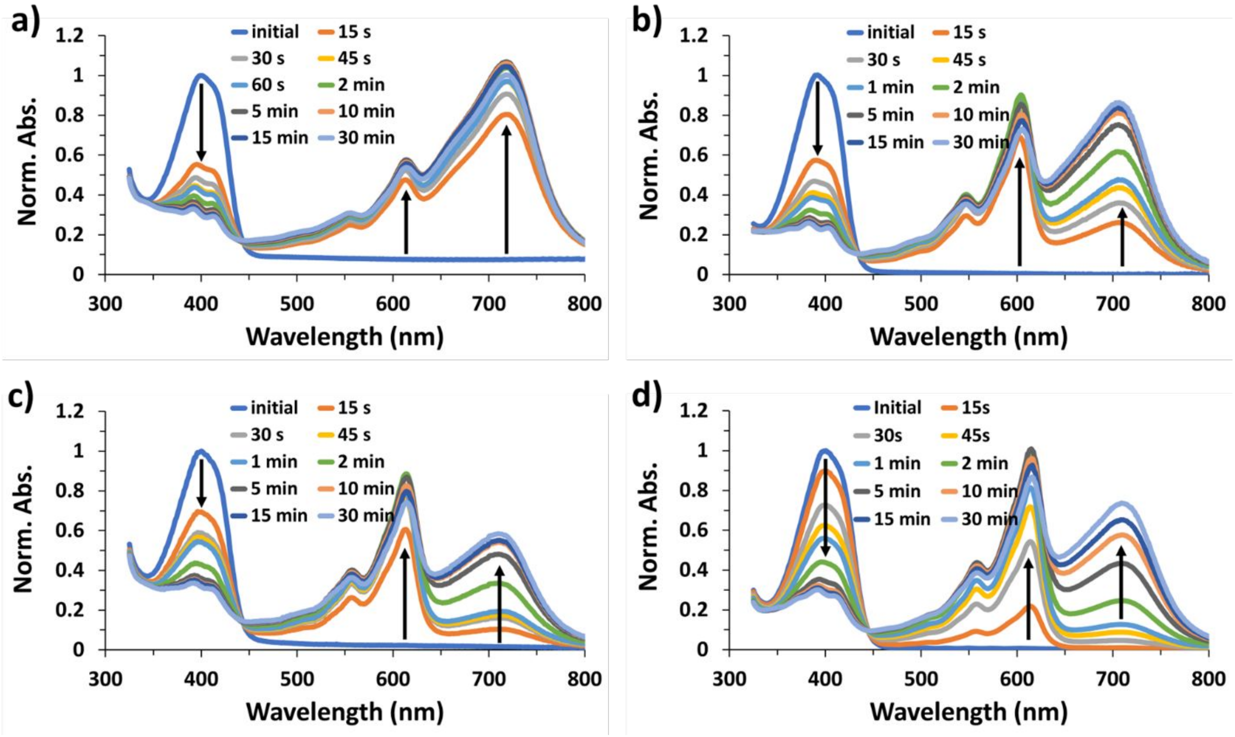

Figure S4: Photochromism of PVA/Borax films with different TTz (wt.%) concentrations, a) 0.4%, b) 1.7%, c) 3.4%, d) 5% (wt. %).

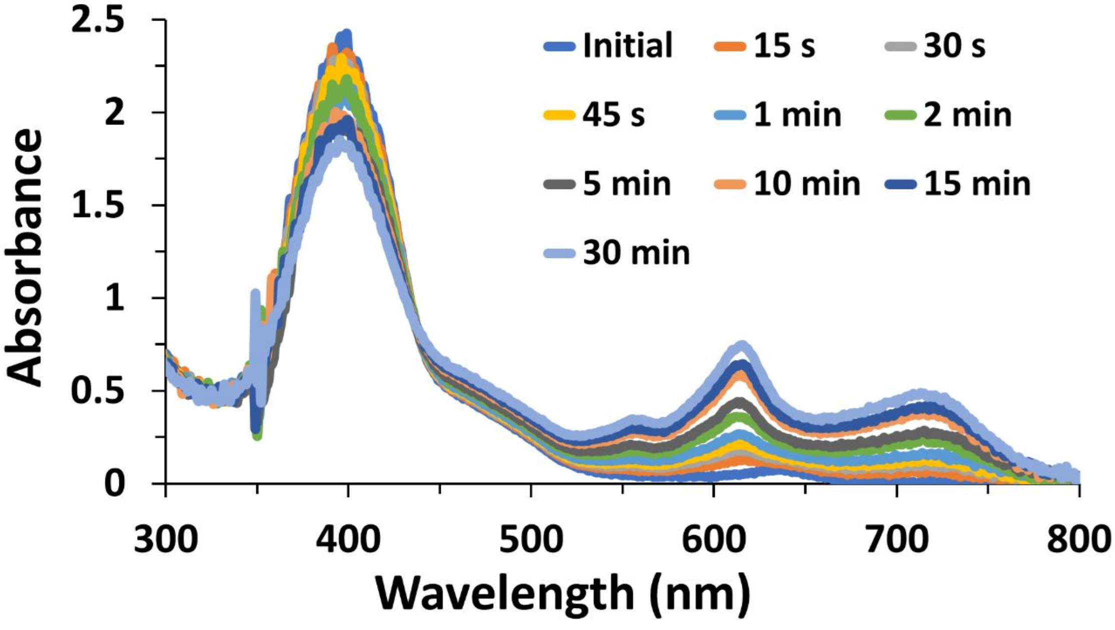

Figure S5: Photochromic absorbance spectra of 1.7 wt. % TTz film with green food dye (tartrazine and brilliant blue FCF).

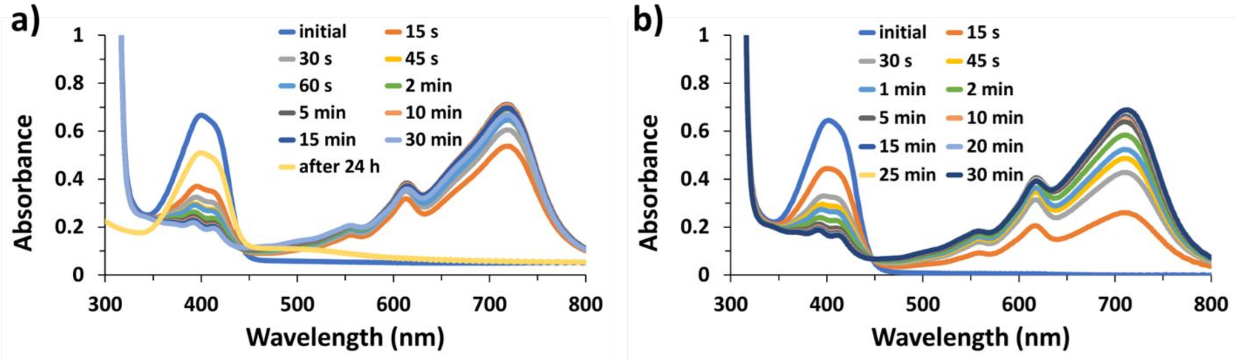

Figure S6: a) Photochromism of 0.4% (wt. %) TTz 14% Borax film dried with normal conditions, b) photochromism of 0.4% TTz 14% Borax film dried in vacuum oven for 72 h.

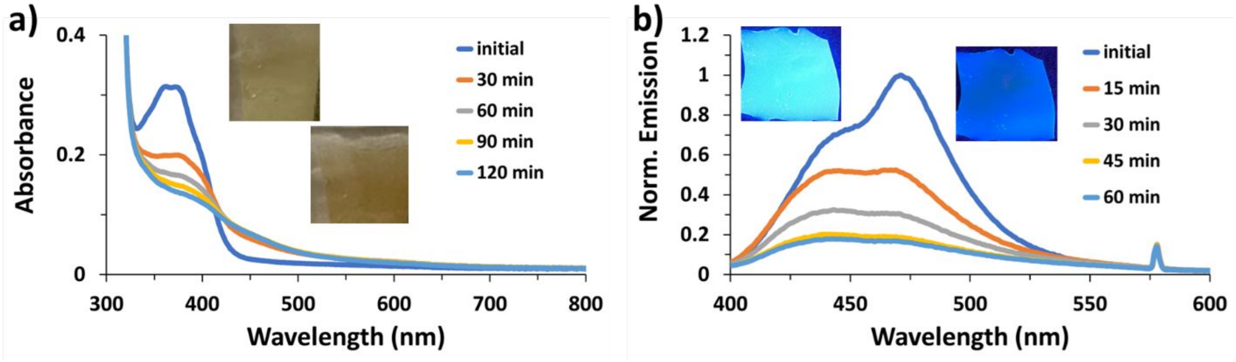

Figure S7: a) Absorbance and b) fluorescence of TTz photodegradation in a PMMA-MAA film, (insets: initial film and film after 60 min illumination).

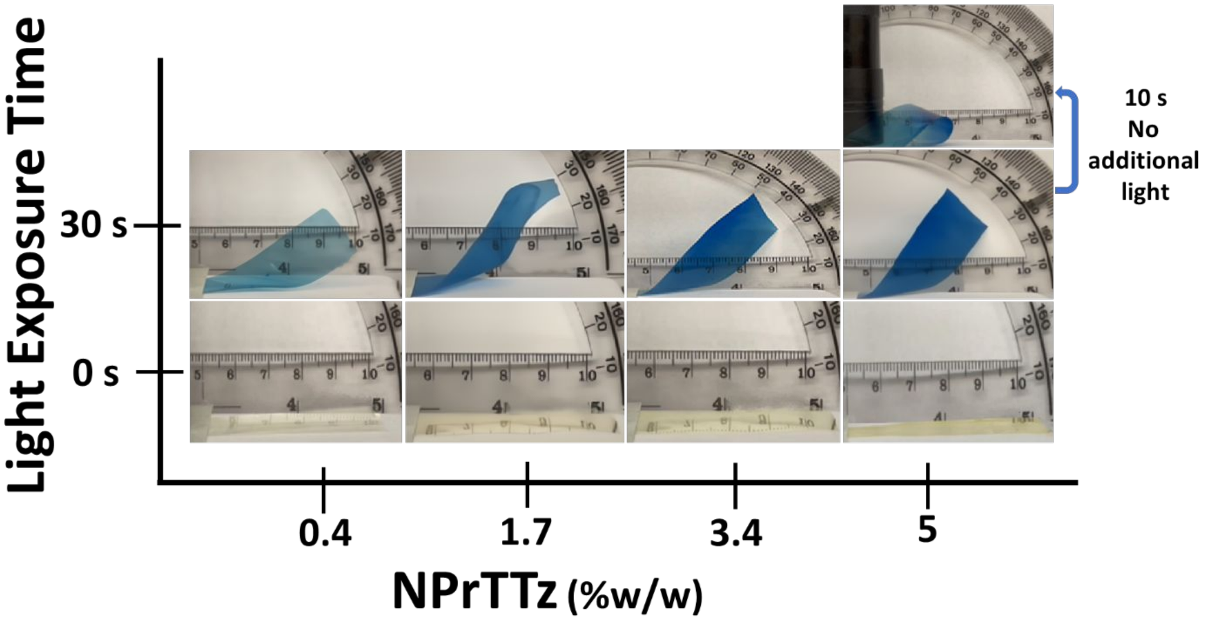

Figure S8: Visual representation of photoactuation of (2 cm x 8 cm) free-standing TTz PVA/borax film before, and after 30 s of 394 nm light irradiation, with increasing NPrTTz concentrations.

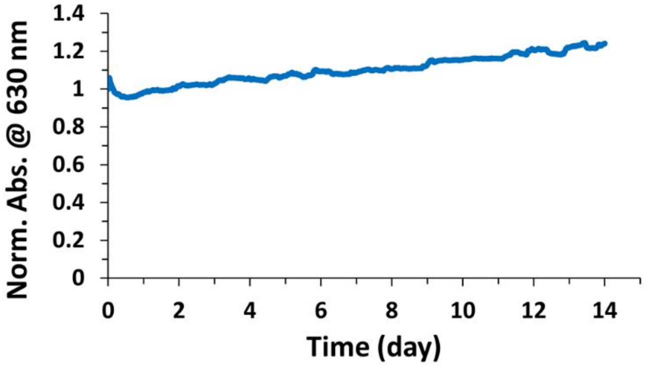

Figure S9: Film absorbance change at 630 nm over 14 d in the ~100 ppm O_2 atmosphere.

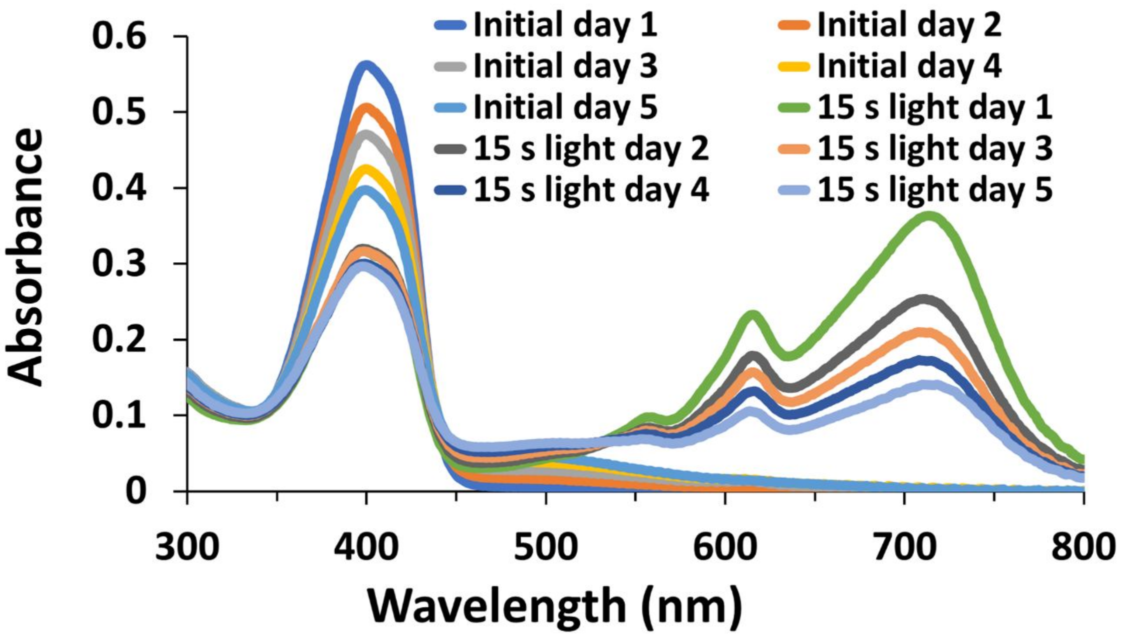

Figure S10: Reversibility of the 0.4% TTz PVA/Borax film at ambient condition cycling yellow to blue for 5 days.

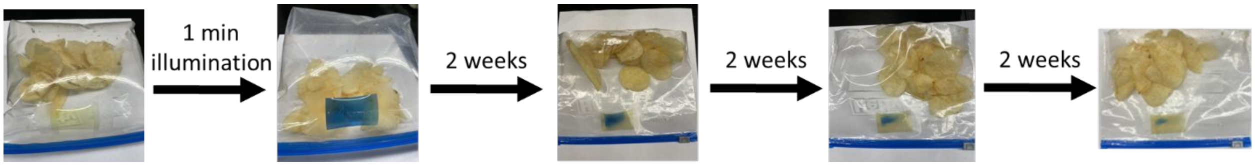

Figure S11: Oxygen sensing TTz film in nitrogen flushed, closed zipper bag.

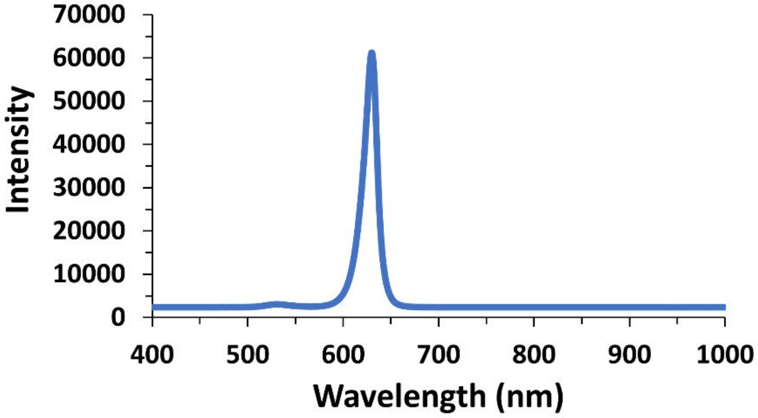

Figure S12: Irradiance spectrum of the red-light used for glovebox atmosphere measurements.

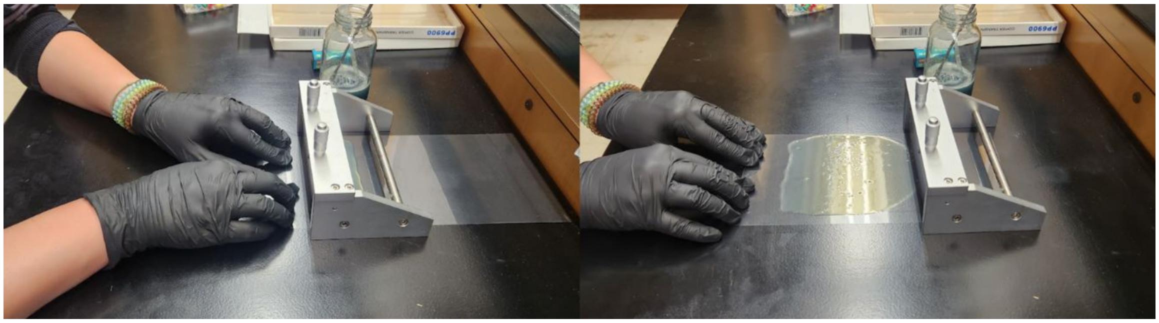

Figure S13: Pictures of the coater and coating the gel onto plastic backing.

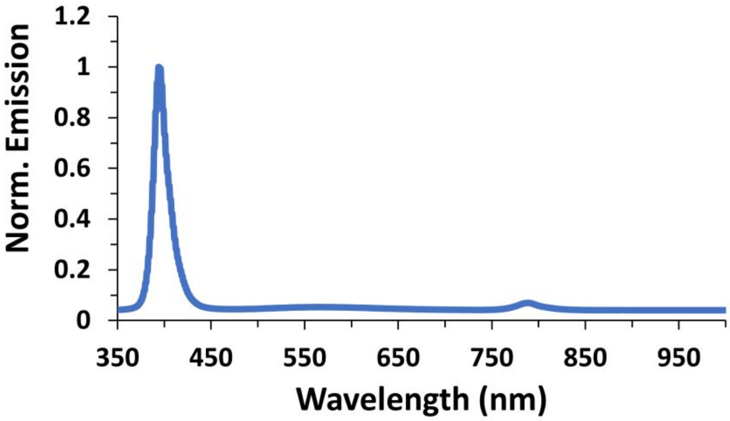

Figure S14: Irradiance spectrum of the uvBeast^{TM} handheld flashlight used for photochromic, photofluorochromic, and photoactuation measurements.
